# Emerging treatment strategies for newly diagnosed diffuse large B-cell lymphoma

**DOI:** 10.1007/s10147-026-03062-7

**Published:** 2026-06-12

**Authors:** Kana Miyazaki

**Affiliations:** Department of Hematology and Oncology, Mie University Graduate School of Medicine, 2-174 Edobashi, Tsu, Mie 514-8507 Japan

**Keywords:** Diffuse large B-cell lymphoma, Cell of origin, Molecular subtype, CAR-T, Bispecific antibody

## Abstract

Despite recent progress in improving patient survival, the outcomes in approximately 40% of patients with diffuse large B-cell lymphoma (DLBCL) remain poor. For many years, R-CHOP (rituximab, cyclophosphamide, doxorubicin, vincristine, and prednisone) has been the standard first-line treatment for DLBCL; however, pola-R-CHP (polatuzumab vedotin, rituximab, cyclophosphamide, doxorubicin, and prednisone) has recently emerged as a new treatment option. In parallel, novel molecularly targeted therapies that target signaling pathways, cellular antigens, and epigenetic regulators associated with high-risk DLBCL are in development. Treatment strategies tailored to the cell of origin or to specific molecular subtypes are also being explored using subtype-specific targeted agents. In addition, agents that have demonstrated efficacy in patients with relapsed or refractory DLBCL—such as the anti-CD19 antibody tafasitamab and immunomodulatory drugs such as golcadomide—are currently under investigation for use in newly diagnosed patients. Immunotherapies, including anti-CD19-chimeric antigen receptor-T therapy and CD20×CD3 bispecific antibodies (BsAbs), have significantly improved outcomes in patients with relapsed or refractory disease and are now under evaluation as early-line therapies. BsAbs can enable more flexible treatment strategies, including combination regimens with cytotoxic or chemotherapy-free approaches. This review summarizes recent advances and ongoing trials that are investigating novel therapeutic strategies for newly diagnosed DLBCL that may soon be incorporated into clinical practice.

## Introduction

Diffuse large B-cell lymphoma (DLBCL) is an aggressive lymphoma that accounts for approximately 40% of all malignant lymphomas. DLBCL includes a heterogeneous group of lymphomas with distinct pathophysiological, genetic, and clinical features. In clinical practice, treatment decisions are also determined on the basis of various factors, such as stage, age, the presence of bulky disease, the International Prognostic Index (IPI) and the National Comprehensive Cancer Network IPI. R-CHOP (rituximab, cyclophosphamide, doxorubicin, vincristine, and prednisone) has long been the standard treatment for DLBCL and has resulted in cures in many patients; however, approximately 30–40% of patients experience refractory disease and relapse [1]. Advances in molecular genetics have improved our understanding of the pathogenesis of DLBCL and have facilitated the development of novel targeted therapies and immunotherapies. More recently, the POLARIX study demonstrated the efficacy of pola-R-CHP (polatuzumab vedotin, rituximab, cyclophosphamide, doxorubicin, and prednisone), which has emerged as a new standard option for previously untreated CD20-positive DLBCL [2]. In addition, the development of immunotherapies such as anti-CD19-chimeric antigen receptor (CAR)-T therapy and CD20×CD3 bispecific antibodies—including epcoritamab, glofitamab, mosunetuzumab, and odronextamab—has significantly changed the treatment landscape for relapsed or refractory (R/R) DLBCL. Novel regimens that incorporate these agents into frontline therapy are currently under investigation, and chemotherapy-free approaches are being explored for elderly and frail patients who are ineligible for intensive cytotoxic chemotherapy. This review summarizes recent advances in the treatment of previously untreated DLBCL and highlights ongoing clinical trials and emerging therapies that may shape future standards of care (Table 1).

**Table 1:** Novel therapeutic agents currently under clinical trials for newly diagnosed DLBCL

Type	Name	Target
Antibody-drug conjuate	Polatuzumab vedotin	CD79b
	Zilovertamab vedotin	ROR1
Antibody	Tafasitamab	CD19
Immunomodulator	Golcadomide	Cereblon
Checkpoint inhibitor	Nivolumab	Programmed cell death protein 1
	Pembrolizumab	Programmed cell death protein 1
	Atezolizumab	Programmed death-ligand 1
Bispecific antibody	Epcoritamab	CD20/CD3
	Glofitamab	CD20/CD3
	Odronextamab	CD20/CD3
	Mosunetuzumab	CD20/CD3
Chimeric antigen receptor T-cell	Axicabtagene ciloleucel	CD19

## Therapeutic approaches based on cell of origin

Gene expression profiling classifies genes into activated-B-cell-like (ABC) and germinal center-like (GCB) subtypes according to the cell of origin [3]. Compared with GCB DLBCL, ABC DLBCL is characterized by nuclear factor κB (NF-κB) pathway activation and inferior survival [4]. To identify more effective therapeutic strategies, a randomized trial was conducted in which R-CHOP was compared with other therapeutic agents that target key molecules in signaling pathways associated with ABC DLBCL. This strategy aims to improve outcomes for all patients with DLBCL by improving the prognosis of ABC DLBCL.

Randomized phase III trials that have evaluated the addition of novel agents to R-CHOP have generally failed to yield a survival benefit. These include the REMoDL-B trial [5], which used R-CHOP combined with bortezomib; the PHOENIX trial [6], which investigated the Bruton’s tyrosine kinase (BTK) inhibitor ibrutinib; and the ROBUST trial [7], which assessed lenalidomide in combination with R-CHOP. However, subgroup analyses suggested a potential benefit of the addition of novel agents to R-CHOP for ABC DLBCL. Long-term follow-up of patients included in the REMoDL-B trial revealed that the addition of bortezomib to R-CHOP improved progression-free survival (PFS) in the ABC and molecular high-grade subgroups, while an additional overall survival (OS) benefit was observed in patients with the ABC subtype [8]. The addition of bortezomib improved the 5-year PFS and OS by 15% and 13%, respectively, in ABC DLBCL patients. In a subgroup analysis of the PHOENIX trial, compared with R-CHOP, ibrutinib plus R-CHOP resulted in significantly longer event-free survival (EFS), PFS, and OS in patients aged < 60 years with ABC DLBCL [9].

Targeted therapeutic strategies for specific DLBCL subtypes are under active investigation (Table 2). Acalabrutinib, a second-generation BTK inhibitor with enhanced BTK selectivity, is currently under evaluation in combination with R-CHOP for use in the patients with newly diagnosed non-GCB DLBCL in the ongoing phase III ESCALADE trial (NCT04529772). Zanubrutinib, another highly selective BTK inhibitor, is also under investigation in combination with pola-R-CHP in newly diagnosed non-GCB DLBCL with extranodal disease (NCT06468943). In addition, zanubrutinib plus lenalidomide and rituximab (ZR2) showed promise in elderly patients (≥ 75 years) with newly diagnosed DLBCL (NCT04460248), with complete response (CR) rates of 65.0% and 2-year PFS and OS rates of 67.1% and 82.4%, respectively [10].

**Table 2:** Ongoing phase III trials of targeted therapies in newly diagnosed DLBCL

Trial (Reference)	Clinical trial identifier	Phase	Number	Trial	Patients	Primary endpoint	Outcomes
ESCALADE	NCT04529772	III	600	Acalabrutinib-R-CHOP × 6 vs. R-CHOP × 6	DLBCL (non-GCB by GEP) Age 18–75 yrs ECOG PS 0–2 Stage II–IV IPI 1–5	PFS	NA
ARCHED [13]	NCT05820841	III	330	Acalabrutinib-R-miniCHOP × 6 vs. R-miniCHOP × 6	DLBCL, PMBCL, leg type, IVLBCL, EBV-positive, HHV8 positive, GZL, HGBCL, FL3B, t-iNHL, TCHRLBCL, ALK-positive LBCL, LBCL with IRF4 rearrangement Age > 80 y or 61–80 yrs with ineligible for full-dose R-CHOP	PFS	NA
PEARL [14]	NCT05179733	III	280	Zanubrutinib-R–lenalidomide × 6 vs. R-miniCHOP × 6	LBCL Age ≥ 80 year with ineligible for anthracycline-based therapy or 70–79 yrs with ineligible for full-dose R-CHOP ECOG PS 0–3	PFS	ZR2 vs. R-miniCHOP CRR: 54.9% vs. 52.1%
GUIDANCE-02	NCT05351346	III	1100	R-CHOP-X vs. R-CHOP	DLBCL ECOG PS 0–2 IPI 2–5 or 1 with bulky disease	PFS	NA
BELIEVE-01	NCT05234684	III	150	Orelabrutinib-R-CHOP × 6 vs. R-CHOP × 6	CD20-positive DLBCL Age 18–80 yrs ECOG PS 0–2 Stage I bulky (> 7.5 cm)–IV IPI 2–5	PFS CRR	NA
ENGINE-1 [19]	NCT03263026	III	256	Enzastaurin-R-CHOP × 6 vs. R-CHOP × 6	CD20-positive DLBCL, HGBCL (DH/TH), HGBCL, NOS Age ≥ 18 yr ECOG PS 0–2 IPI 3–5	OS in in subjects who possess the *DGM1* biomarker	NA
POLAR BEAR [25]	NCT04332822	III	300	Pola-R-miniCHP × 6 vs. R-miniCHOP × 6	CD20-positive DLBCL Age ≥ 80 yrs or 75–80 yrs with frail ECOG PS 0–3	PFS	Pola-R-miniCHP vs. R-miniCHP > G1 gastrointestinal toxicity: 31% vs. 16% G 1–2 peripheral neuropathy: 8 pts vs. 10 pts
waveLINE-010 [30]	NCT06717347	III	1046	Zilovertamab vedotin-R-CHP × 6 vs. R-CHOP × 6	DLBCL Age ≥ 18 years Stage II–IV ECOG PS 0–2	PFS	NA
GOLSEEK-1	NCT06356129	III	850	Golcadomide-R-CHOP versus R-CHOP	DLBCL, NOS, HGBCL, HGBCL, NOS, TCHRLBCL, EBV-positive Age 18–80 yrs Stage II–IV IPI 3–5 or 1 or 2 with LDH (> 1.3 ULN) and/or bulky disease (≥ 7 cm)	PFS	NA
SWOG 1918	NCT04799275	II/III	422	Oral azacitidine-R-miniCHOP × 5 + R-miniCHOP ×1 vs. R-miniCHOP × 6	DLBCL, NOS, TCHRBCL, PMBCL, leg type, IVLBCL, EBV-positive, HHV8 positive, t-iNHL, FL3B Age ≥ 75 yrs Stage II bulky–IV ECOG PS 0–2	PFS (phase II) OS (phase III)	NA
DEB	NCT04231448	III	423	Tucidinostat-R-CHOP × 6 vs. R-CHOP × 6	Double expresser lymphoma Age 18–80 yrs ECOG PS 0–2 IPI 2–4	EFS	Tucidinostat-R-CHOP vs. R-CHOP: Median EFS: 35.3 mo vs. 26.4 mo 2-yr EFS: 60.3% vs. 50.5% CRR: 73.0% vs. 61.8%

CRR, complete response rate; DH/TH, double hit/triple hit; DLBCL, diffuse large B-cell lymphoma; ECOG, Eastern Cooperative Oncology Group; EBV, Epstein-Barr virus; EFS, even-free survival; FL3G, follicular lymphoma grade 3B; G, grade; GCB, germinal centre B-cell-like, GEP, gene expression profiling; GZL, gray zone lymphoma; HGBCL, high-grade B-cell lymphoma; HHV8, human herpes virus 8; IPI, International Prognostic Index; IVLBCL, intravascular large B-cell lymphoma; LBCL, large B-cell lymphoma; leg type, primary cutaneous large B-cell lymphoma, leg type; NA, not available; NOS, not otherwise specified; OS, overall survival; PMBCL, primary mediastinal large B-cell lymphoma; pola-R-miniCHP, polatuzumab vedotin, rituximab, cyclophosphamide, doxorubicin, and prodnisone; mo; months; PFS, progression-free survival; PS, performance status; pola, polatuzumab vedotin; R, rituximab; R-CHP, rituximab, cyclophosphamide, doxorubicin, and prodnisone; R-CHOP, rituximab, cyclophosphamide, doxorubicin, vincristine, and prodnisone; TCHRBCL, T-cell/histiocyte-rich large B-cell lymphoma; tFL, transformed follicular lymphoma; t-iNHL, transformed indolent non-Hodgkin lymphoma; ULN, upper limit of normal; vs., versus; yr, year

The incidence of ABC DLBCL increases with age and accounts for approximately 60–70% of DLBCL patients aged ≥ 80 years [11, 12]. The ongoing phase III ARCHED trial (NCT05820841) is evaluating acalabrutinib plus R-miniCHOP versus R-miniCHOP in patients aged > 80 years or > 60 years who are ineligible for full-dose R-CHOP [13]. Similarly, the phase III PEARL trial (NCT05179733) compared six cycles of ZR2 with six cycles of R-miniCHOP in elderly or frail patients with newly diagnosed DLBCL; preliminary results in 284 patients revealed comparable CR rates (54.9% vs. 52.1%) [14].

### Therapeutic approaches based on molecular subtype

The LymphGen classification categorizes DLBCL into six genetic subtypes: MCD (*MYD88*^L265P^ and *CD79B* mutations), BN2 (*BCL6* fusions and *NOTCH2* mutations), N1 (*NOTCH1* mutations), EZB (*EZH2* mutations and *BCL2* translocations), A53 (*TP53* mutations), and ST2 (JAK/STAT pathway alterations, including *TET2* and *SGK1* mutations). Among these subtypes, ST2, EZB, and BN2 are associated with relatively favorable outcomes, whereas MCD and N1 are linked to a worse prognosis [15, 16]. The phase II GUIDANCE-01 trial evaluated a genomics-guided strategy (R-CHOP-X) in which targeted agents were selected according to molecular subtype (e.g., ibrutinib for MCD/BN2, lenalidomide for N1/DLBCL-NOS, tucidinostat for EZ, and decitabine for *TP53*-mutant disease) [17]. Compared with R-CHOP, R-CHOP-X significantly improved the CR rate (88% vs. 66%), overall response rate (ORR) (92% vs. 73%), and the 2-year PFS (88% vs. 63%) and OS (94% vs. 77%). These findings suggest the potential of genomics-guided personalized therapy in DLBCL, and a confirmatory phase III GUIDANCE-02 trial (NCT05351346) is ongoing in China (Table 2). Orelabrutinib, a highly selective covalent BTK inhibitor that penetrates the blood–brain barrier, is under investigation in the phase III BELIEVE-01 trial (NCT05234684), which is comparing orelabrutinib plus R-CHOP versus R-CHOP for newly diagnosed DLBCL (Table 2). Enzastaurin, an oral PKC-β and PI3K/AKT pathway inhibitor, failed to improve PFS as a maintenance therapy after R-CHOP [18]. The phase III ENGINE1 trial (NCT03263026) is evaluating enzastaurin plus R-CHOP versus R-CHOP in high-risk DLBCL (IPI 3–5), with OS in patients harboring the *DGM1* polymorphism as the primary endpoint (Table 2) [19].

### Antibody-drug conjugates

Polatuzumab vedotin (pola), a CD79B-directed antibody–drug conjugate that delivers monomethyl auristatin E (MMAE) [20–22], has demonstrated a clinical benefit as a frontline therapy for DLBCL. In the phase III POLARIX trial, compared with R-CHOP, pola-R-CHP significantly improved PFS in patients aged 18–80 years with IPI of 2–5, with a sustained benefit at long-term follow-up (5-year PFS 64.9% vs. 59.1%) [2, 23]; thus, pola-R-CHP was established as a standard treatment option for this population. In a subgroup analysis of POLARIX, compared with GCB-type DLBCL, pola-R-CHP offered a substantially greater benefit to patients with ABC-type DLBCL [24]. The Nordic Lymphoma Group conducted a randomized phase III POLAR BEAR trial (NCT04332822) that compared six cycles of R-miniCHOP with pola-R-mini-CHP in newly diagnosed DLBCL patients aged ≥ 80 years and in frail patients aged 75–80 years (Table 2) [25]. With a median follow-up time of 11.4 months, the safety results revealed no difference in grade 3–4 hematologic toxicity or peripheral neuropathy between the groups, although gastrointestinal adverse events were more frequent in the group treated with pola-R-mini-CHP.

Zilovertamab vedotin (ZV) is an antibody–drug conjugate that targets tyrosine kinase-like orphan receptor 1 (ROR1), which is frequently overexpressed on the surface of lymphoma cells and is conjugated to MMAE [26]. ZV binding to tumor cell ROR1 results in rapid internalization, trafficking to lysosomes, antibody–drug conjugate cleavage, and MMAE release. ZV has demonstrated a tolerable safety profile and promising antitumor activity in R/R non-Hodgkin lymphoma, including DLBCL [27]. The phase II waveLINE07 trial (NCT05406401) evaluated the safety and efficacy of 6–8 cycles of ZV plus R-CHP in newly diagnosed DLBCL patients (Table 2). Preliminary results after a median follow-up time of 26.6 months revealed an overall CR rate of 97.2% (35/36) [28] and a 24-month CR rate of 84%, while the median duration of CR was not reached. A randomized phase II waveLINE-011 trial (NCT06890884) of ZV plus R-CHP versus pola-R-CHP in newly diagnosed GCB DLBCL patients with PS scores of 0–2 and IPIs of 2–5 is ongoing [29]. In addition, the randomized phase III waveLINE-010 trial (NCT 06717347) comparing ZV plus R-CHP with R-CHOP in newly diagnosed DLBCL patients with PS scores of 0–2 and IPI of 2–5 has been initiated (Table 2) [30].

### Tafasitamab/lenalidomide

Tafasitamab, a humanized anti-CD19 monoclonal antibody with a modified Fc region that enhances antibody-dependent cellular cytotoxicity, antibody-dependent cellular phagocytosis and direct cytotoxicity [31, 32], is approved in Europe and the United States for the treatment of R/R DLBCL. The randomized phase III FrontMIND trial (NCT04824092) evaluated tafasitamab plus lenalidomide in combination with R-CHOP versus R-CHOP alone in patients with newly diagnosed DLBCL [33]. This study demonstrated a significant improvement in PFS with tafasitamab and lenalidomide combined with R-CHOP [34]. This combination may represent a promising strategy to address the 40% relapse rate observed with R-CHOP.

### Immunomodulators

The development of next-generation immunomodulatory drugs is also underway. Golcadomide binds to the cereblon E3 ligase with higher affinity than does lenalidomide and promotes the recruitment of the Ikaros/Aiolos complex to the ubiquitination ligase complex, which leads to its ubiquitination and proteasomal degradation. The randomized phase III GOLSEEK-1 trial (NCT06356129) is evaluating golcadomide plus R-CHOP versus R-CHOP in approximately 850 patients with newly diagnosed LBCL, including those with DLBCL, NOS, and high-grade B-cell lymphoma with *MYC* and *BCL2* rearrangements (Table 2).

### Checkpoint inhibitors

The efficacy of PD-1 immune checkpoint inhibitors, such as nivolumab and pembrolizumab, as monotherapies in patients with R/R DLBCL appears to be limited. In a single-arm phase II trial of nivolumab in patients with R/R DLBCL who were ineligible for autologous hematopoietic stem cell transplantation (AHSCT) or who had relapsed after AHSCT, the ORR was 3% in the AHSCT-ineligible group and 10% in the post-AHSCT relapse group. Similarly, a phase II trial of pembrolizumab as a consolidation therapy after AHSCT in patients with DLBCL failed to meet its primary endpoint despite an 18-month PFS rate of 59%. Immune checkpoint inhibitors have also been evaluated in combination with chemoimmunotherapy. In the single-arm phase II HOVON-152 trial, nivolumab consolidation therapy after 1 cycle of R-CHOP followed by 5 cycles of DA-EPOCH-R induction in newly diagnosed high-grade B-cell lymphoma patients with *MYC* and *BCL2* and/or *BCL6* rearrangements resulted in a CR rate of 61% (20/33) [35]. In addition, a phase I trial of pembrolizumab plus R-CHOP in newly diagnosed DLBCL or grade 3b follicular lymphoma (FL) demonstrated a manageable safety profile and a durable response (5-year PFS 71% and OS 83%) [36]. Atezolizumab, a PD-L1 inhibitor, has also been investigated in combination with R-CHOP in previously untreated FL or DLBCL, and encouraging clinical activity was shown in a phase Ib/II study [37].

### Bispecific antibodies

Bispecific antibodies, which contain two antigen-binding domains that simultaneously target tumor cells and immune cells, typically engage CD3 on T cells and a tumor-associated antigen. This interaction promotes T-cell activation, proliferation, and cytotoxicity through the release of granzymes and perforin, leading to tumor cell death. Recent IgG-like bispecific antibodies include an Fc region, which provides a longer half-life than blinatumomab, as this antibody lacks an Fc region. Many of these bispecific antibodies incorporate Fc-silencing mutations to reduce unintended immune activation and effector functions [38]. Several CD3×CD20 bispecific antibodies have demonstrated promising efficacy in R/R DLBCL and are already in clinical use. Clinical trials are currently evaluating their combination with chemotherapy in patients with newly diagnosed DLBCL (Table 3).

**Table 3:** Prospective clinical trials evaluating immunotherapy in newly diagnosed DLBCL

Trial (References)	Clinical trial identifier	Phase	Number	Trial	Patients	Outcomes	Toxicities (CRS)	Toxicities (ICANS)
EPCORE NHL-2 (Arm 1) [39, 40]	NCT04663347	Ib/II	47	Epcoritamab + R-CHOP x 6 + epcoritamab maintenance (1 year)	CD20-positive DLBCL Age ≥ 18 yr ECOG PS 0–2 IPI 3–5	ORR: 98% CRR: 85%	G1 45% G2 11% G3 4%	G1, *n*=1 G2, *n* = 1
EPCORE DLBCL-2	NCT05578976	III	900	Epcoritamab + R-CHOP x 6 vs. R-CHOP x 6	LBCL (DLBCL, NOS, HGBCL (DH/TH), TCRLBCL, EBV-positive, FL3B) Age 18–79 yrs ECOG PS 0–2 IPI 2–5 (IPI 2 capped at 35%)	NA	NA	NA
EPCORE NHL-5 (Arm 3) [41]	NCT05283720	Ib/II	37	Epcoritamab + pola-R-CHP x 6 + epcoritamab x 2	CD20-positive DLBCL Age ≥ 18 yr ECOG PS 0–2 IPI 2–5	ORR: 100% (27/27) CRR: 89% (24/27)	G1-2: 35% (6/17)	NA
EPCORE NHL-2 (Arm 8) [42]	NCT04663347	Ib/II	28	Epcoritamab-R-miniCHOP x 6 + epcoritamab x 2	CD20-positive DLBCL (DLBCL, NOS, HGBCL (DH/TH), TCRLBCL, FL3B) Age ≥ 75 year with ineligible for full-dose R-CHOP or ≥ 65 year with comorbidities	ORR: 89% (25/28) CRR: 82% (23/28) 1-yr PFS: 88% 1-yr OS: 96%	G1 25% G2 29%	None
EPCORE NHL-3 (Arm A) [43]	NCT05660967	II	45	Epcoritamab (up to 1 year)	CD20-positive LBCL, stage II-IV Age ≥ 80 year with ineligible for anthracycline-based therapy or ≥ 75 year with comorbidities ECOG PS 0–2 (3 if attributable to lymphoma)	ORR: 74% (29/39) CRR: 64% (25/39)	G1 36% G2 27% G3 5%	G1 9% G2 5% G3 2%
GO43075 [45]	NCT04980222	II	24	Glofitamab-R-CHOP	ct DNA high risk LBCL (DLBCL, NOS, HGBCL (DH/TH), tFL) ECOG PS 0–2 IPI 2–5	ORR: 93.3% (14/15) CRR: 80% (12/15)	G1 16.7% G2 4.2%	None
COALITION [46]	NCT04914741	II	81	Glofitamab-R-CHOP (A) vs. glofitamab-pola-R-CHP (B)	LBCL (DLBCL, NOS, HGBCL Age 18–65 yrs IPI ≥ 3, NCCN-IPI ≥ 4 or HGBCL (DH/TH) ECOG PS 0–3 (3 if attributable to lymphoma)	A vs. B: ORR: 98% vs. 98% CRR: 68% vs. 80% 2-yr PFS: 86% vs. 86% 2-yr OS: 92% vs. 91%	A: G1 18% G2 3% B: G1 20% G2 3%	None
Dana-Farber Cancer Institute	NCT05800366	II	40	Pola-R-CHP x 2 + glofitamab-pola-R-CHP x 4 + glofitamab x 2	CD20-positive DLBCL Age ≥ 18 yr ECOG PS 0–2 IPI 2–5	NA	NA	NA
GRAIL	NCT06050694	II	40	Favourable ctDNA and PET2 negative: Pola-R-CHP x 4 + R x 2 Unfavourable ctDNA and/or PET2 positive: Pola-R-CHP x 2 + glofitamab-pola-R-CHP x 4	CD20-positive DLBCL Age ≥ 18 yr ECOG PS 0–2	NA	NA	NA
GLORY	NCT06765317	II	42	PET2-negative: glofitamab-pola x 2 + glofitamab-pola-R-miniCHP x 4 PET2-positive: glofitamab-pola x 2 + glofitamab-pola-RminiCHP x 6	DLBCL, HGBCL, tDLBCL Age ≥ 80 year or 65–79 yrs with unfit/frail Stage II bulky–IV ECOG PS 0–2	NA	NA	NA
Dickinson, et al. [48]	NCT03467373	Ib	24	pola-R-CHP x 1 + glofitamab-pola-R-CHP x 5	Part I: r/r NHL Part II: CD20-positive DLBCL Age ≥ 18 yr ECOG PS 0–2	ORR: 100% (17/17) CRR: 76.5% (13/17)	G1 13%	Neurologial AE: G1-2 42% G3, 4%
SKYGLO [47]	NCT06047080	III	1,130	pola-R-CHP x 1 + Glofitamab-pola-R-CHP x 5 + glofitamab x 2 vs. pola-R-CHP x 6 + R x 2	LBCL (exclusion: tFL, FL3B) Age 18–80 yrs ECOG PS 0–2 IPI 2–5	NA	NA	NA
AGMT-NHL-16/GLA2022-10/IKF062* (489)	EudraCT No.: 2022-003398-51	II	80	Obinutuzumab-pola-glofitamab x 6 + glofitamab x 6	LBCL (DLBCL, NOS, HGBCL Age ≥ 60 year with ineligible for full-dose R-CHOP ECOG PS 0–3 (3 if attributable to lymphoma)	ORR: 90% (17/17) CRR: 81% (13/17) 1-yr PFS: 85% 1-yr OS: 90%	First 10 patients: G1 30%	First 10 patients: none
OLYMPIA-3, Part 1 A [51]	NCT06091865	III	22	Dose escalation: Odronextamab-R-CHOP x 6 vs. R-CHOP x 6	CD20-positive DLBCL Age ≥ 18 yr ECOG PS 0–2 IPI 3–5 or IPI 2–5 (non-GCB) or HGBCL	DL1: ORR 77.8%, CRR 66.7% DL2: ORR 100%, CRR 100%	G1 (DL1, 11.1%; DL2, 69.2%)	None
OLYMPIA-3	NCT06091865	III	904	Odronextamab-R-CHOP x 6 vs. R-CHOP x 6	CD20-positive DLBCL (DLBCL, NOS, HGBCL, FL3B) Age ≥ 18 year, stage II–IV ECOG PS 0–2 IPI 2–5	NA	NA	NA
Olszewski, et al. [53]	NCT03677141	II	40	Mosunetuzumab-CHOP x 6	DLBCL ECOG PS 0–2 IPI 2–5	ORR: 87.5% CRR: 85.0% 2-yr PFS: 65.4% 2-yr EFS: 60%	G1 45% G2 15%	2.50%
GO40515 [54]	NCT03677141	Ib/II	62 (2:1)	Pola-mosunetuazumab IV-CHP x 6 vs. pola-R-CHP x 6	DLBCL ECOG PS 0–2 IPI 2–5	Pola-M-CHP vs. Pola-R-CHP: 2-yr PFS: 70.8% vs. 81.8% 2-yr OS: 86.3% vs. 86.4%	Pola-M-CHP: G1 52.6% G2 13.2% G3 2.6%	Any G 7.9% G1, *n* = 1 G3, *n* =1 G5, *n* = 1
MorningSun [55]	NCT05207670	II	49 (total 320)	Mosunetuzumab SC x 8 (Max 17 cycles)	B-NHL Age ≥ 80 year or 65–79 yrs with ineligible for chemoimmunotherapy	1-yr PFS: 68.8% 1-yr OS: 77.2%	G1 10.2% G2 2%	None
GO40554 (Cohort B)	NCT03677154	I/II	54	Mosunetuzumab IV x 8 (Max 17 cycles)	B-NHL Age ≥ 80 year or 65–79 yrs with ineligible for chemoimmunotherapy	ORR: 43% CRR: 35% 1-yr PFS: 39%	Any G 26% G1, *n* = 10 G2, *n* = 4	None
GO40554 (Cohort C) [56]	NCT03677154	II	108	Mosunetuzumab SC-pola	Unfit/frail DLBCL Age ≥ 80 year or 65–79 yrs with ineligible for full-dose chemoimmunotherapy	ORR: 55% (31/56) CRR: 45% (25/56)	Any G 30%	NA
ZUMA-12 [61, 62]	NCT03761056	II	40	Axi-cel	LBCL, IPI 3-5 or HGBCL (DH/TH), HGBCL-NOS PET positivitiy after chemoimmunotherapy x 2	ORR: 92% CRR: 86% 36-mo EFS: 73.0% 36-mo PFS: 75.1% 36-mo OS: 81.1%	G1 68% G2 25% G3 8%	Neurologial AE: ≥G3 Confusional state: 5% Encephalo-pathy: 15%
ZUMA-23 [63]	NCT05605899	III	300	Axi-cel vs. R-CHOP/DA-EPOCH-R	LBCL (DLBCL, NOS, HGBCL (DH/TH), HGBCL-NOS), tFL, tMZL ECOG PS 0-2 Stage III/IV IPI 4–5	NA	NA	NA

AE, adverse event; axi-cel, axicabtagene ciloleucel; ct, circulating tumor; CRR, complete response rate; CRS, cytokine release syndrome; DA-EPOCH-R, dose-adjusted etoposide, predonison, vincristine, cyclophosphamide, and doxorubicin; DH/TH, double hit/triple hit; DL, dose level; DLBCL, diffuse large B-cell lymphoma; ECOG, Eastern Cooperative Oncology Group; EFS, even-free survival; FL3G, follicular lymphoma grade 3B; G, grade; IV, intravenous; HGBCL, high-grade B-cell lymphoma; ICANS, immune effector cell-associated neurotoxicity syndrome; IPI, International Prognostic Index; NOS, not otherwise specified; ORR, overall response rate; OS, overall survival; pola-R-CHP, polatuzumab vedotin, rituximab, cyclophosphamide, doxorubicin, and prodnisone; LBCL, large B-cell lymphoma; mo; months; NA, not available; NCCN, National Comprehensive Cancer network; NHL, non-Hodgkin lymphoma; PET, positron emission tomography; PFS, progression-free survival; PS, performance status; pola, polatuzumab vedotin; R, rituximab; R-CHOP, rituximab, cyclophosphamide, doxorubicin, vincristine, and prodnisone; r/r; relapsed/refractory; SC, subcutaneous; tFL, transformed follicular lymphoma; tMZL, transformed marginal zone lymphoma; vs., versus; yr, year

Epcoritamab is a subcutaneous CD3×CD20 bispecific antibody that has demonstrated promising efficacy and safety in R/R DLBCL and is already used in clinical practice after ≥ 2 prior lines of therapy. In the phase Ib/II EPCORE NHL-2 trial (Arm 1, NCT04663347), epcoritamab plus R-CHOP was evaluated in patients with newly diagnosed CD20 + DLBCL with high-risk disease (IPI 3–5) [39, 40]. With a median follow-up of 38.8 months, the ORR and the CR rate were 98% and 85%, respectively. Grade 3 cytokine release syndrome (CRS) events occurred in 4% of the patients, and immune effector cell-associated neurotoxicity syndrome (ICANS) occurred in 2 patients. On the basis of these results, the phase III EPCORE DLBCL-2 trial (NCT05578976) is currently comparing epcoritamab plus R-CHOP with R-CHOP in newly diagnosed DLBCL patients. In addition, the phase Ib/II EPCORE NHL-5 trial (Arm 3, NCT05283720) for newly diagnosed CD20 + DLBCL patients with ECOG PS 0–2 and IPI 2–5 evaluated epcoritamab plus pola-R-CHP and reported an ORR of 100% and a CR rate of 89% [41]. Low-grade CRS events occurred in 6/17 (35%) patients.

Clinical trials that include elderly or frail patients are also ongoing. In the phase Ib/II EPCORE NHL-2 trial (Arm 8, NCT04663347), patients aged ≥ 75 years or ≥ 65 years with comorbidities who were ineligible for full-dose R-CHOP were treated with fixed-duration epcoritamab plus R-mini-CHOP and achieved an ORR of 89% and a CR rate of 82% [42]. A randomized phase 2 EPCORE NHL-3 trial comparing a fixed duration (up to one year) of epcoritamab monotherapy (Arm A, NCT56660967) in untreated CD20 + LBCL patients aged ≥ 80 years or ≥ 75 years with comorbidities who were ineligible for anthracycline-based treatment is ongoing [43]. The ORR was 74% (29/39), and the CR rate was 64% (25/39), with manageable incidences of CRS and ICANS.

Glofitamab is a bivalent CD20-targeting CD3×CD20 bispecific antibody that has shown promising activity in R/R DLBCL [44]. Early studies evaluated its use in a frontline setting in combination with chemotherapy. In a phase II trial that evaluated glofitamab plus R-CHOP in 24 patients with newly diagnosed high-risk LBCL defined by circulating tumor DNA dynamics (NCT04980222) [45], the CR rate was 80%, and the ORR was 93.3%. CRS occurred in 20.8% (5/24) of patients, and no ICANS was observed. In the phase II COALITION trial (NCT04914741), younger patients (≤ 65 years) with newly diagnosed LBCL and at least one high-risk feature (IPI ≥ 3, NCCN-IPI ≥ 4, or rearrangements of *MYC* and *BCL2* and/or *BCL6*) were treated with glofitamab plus R-CHOP or glofitamab plus pola-R-CHP. Both regimens were safely deliverable and achieved high durable response rates [46]. The ORR was identical in both groups (98%), while a higher CR rate was observed with glofitamab plus pola-R-CHP (80%) than with glofitamab plus R-CHOP (68%). The ongoing randomized SKYGLO trial (NCT06047080) is evaluating glofitamab-pola-R-CHP (1 cycle of pola-R-CHP plus 5 cycles of glofitamab-pola-R-CHP plus 2 cycles of glofitamab) versus pola-R-CHP (6 cycles of pola-R-CHP plus 2 cycles of rituximab) in newly diagnosed LBCL patients aged 18–80 years with IPI scores of 2–5 [47]. In the glofitamab plus pola-R-CHP arm, patients are designated to receive one cycle of pola-R-CHP followed by glofitamab in combination with pola-R-CHP during cycles 2–8 using a step-up dosing schedule; this regimen is compared with six cycles of pola-R-CHP. This strategy is based on findings from a phase Ib study (NCT03467377), which suggested that the administration of glofitamab after the tumor burden is reduced may decrease the incidence of CRS [48]. Clinical trials evaluating glofitamab-based regimens in elderly and frail patients are also ongoing. The Austrian Group for Medical Tumor Therapy and the German Lymphoma Alliance conducted a phase II trial (AGMT-NHL-16/GLA2022-10/IKF062) in elderly, frail or otherwise medically unfit patients newly diagnosed with DLBCL who were ineligible for full-dose R-CHOP. In this study, patients were treated with R-Pola-Glo (rituximab, pola, and glofitamab). Among the first 10 treated patients, an excellent safety profile was observed, and of these, three patients had grade 1 CRS and none had ICANS [49]. With a median follow-up of 15 months, the estimated 1-year PFS, EFS, and OS were 85%, 82%, and 90%, respectively [50].

Odronextamab, a CD20×CD3 bispecific antibody, has demonstrated promising efficacy as a monotherapy in patients with R/R DLBCL. The phase III OLYMPIA-3 trial (NCT06091865) is currently underway and seeks to evaluate whether odronextamab plus CHOP is superior to R-CHOP in patients with newly diagnosed DLBCL. In the dose escalation phase of the OLYMPIA-3 trial (Part 1 A), induction therapy with odronextamab plus CHOP resulted in a generally good safety profile [51].

Mosunetuzumab is an off-the-shelf CD20xCD3 T-cell–engaging bispecific antibody that redirects T cells to eliminate B cells. In the phase III SUNMO trial, transplant-ineligible patients with R/R LBCL were randomized at a 2:1 ratio to receive Mosun-Pola (mosunetuzumab and pola) or R-GemOx (rituximab, gemcitabine, and oxaliplatin) [52]. Compared with R-GemOx, the median PFS of the Mosun-Pola group was significantly longer (11.5 vs. 3.8 months) with the median follow-up of 23.2 months. In addition, a phase II study (NCT03677141) evaluated six cycles of mosunetuzumab in combination with CHOP in 40 patients with newly diagnosed DLBCL (PS 0–2; IPI 2–5) [53]. With a median follow-up of 32.0 months, the estimated 2-year PFS and EFS rates were 65.4% and 60.4%, respectively. CRS occurred in 60.0% of patients but was limited to grades 1–2. The results of the phase II study compared mosunetuzumab plus pola-CHP (pola-M-CHP) with pola-R-CHP in newly diagnosed DLBCL (PS 0–2; IPI 2–5) was reported [54]. Pola-M-CHP did not demonstrate a clinical benefit over Pola-R-CHP. Serious adverse events (63.2% vs. 13.6%), and adverse events leading to treatment discontinuation (13.2% vs. 0%) were higher with Pola-M-CHP than Pola-R-CHP, respectively. Mosunetuzumab has also been investigated in elderly patients. Interim results from the phase II MorningSun trial (NCT05207670) evaluated fixed-duration subcutaneous mosunetuzumab monotherapy in patients with B-cell non-Hodgkin lymphoma aged ≥ 80 years or aged 65–79 years who were considered ineligible for chemoimmunotherapy. The 1-year PFS rate was 68.8%, and the 1-year OS rate was 77.2% [55]. A phase Ib/II trial (NCT03677154) evaluated Mosun-Pola in patients aged ≥ 80 years or aged 65–79 years who were ineligible for full-dose chemoimmunotherapy [56]. Preliminary results revealed an ORR of 55% (31/56) and a CR rate of 45% (25/56) after a median follow-up of 7.5 months. A manageable safety profile was also reported.

### Anti-CD19 chimeric antigen receptor T-cell therapy

Clinical trials demonstrating the high efficacy and manageable safety profiles of anti-CD19 CAR-T-cell therapy for R/R DLBCL have been reported [57]. CAR-T-cell therapy has already been added as a third-line therapeutic option for R/R DLBCL. Moreover, axicabtagene ciloleucel (axi-cel) and lisocabtagene maraleucel have become the standard treatment options for patients with DLBCL who are refractory to disease or who have relapsed after first-line therapy within one year after they achieve CR [58–60]. The development of CAR-T-cell therapy as a first-line treatment for newly diagnosed DLBCL has recently begun (Table 3). In the single-arm phase II ZUMA-12 trial (NCT03761056), 40 patients with high-risk large B-cell lymphoma (LBCL) defined by interim PET2 positivity (Deauville score 4–5) after two cycles of chemoimmunotherapy together with either double- or triple-hit lymphoma or IPI scores of 3–5 received axi-cel [61]. The CR rate increased to 86%, with an ORR of 92%, and the 36-month EFS, PFS, and OS were 73.0%, 75.1%, and 81.1%, respectively. Grade ≥ 3 CRS and neurologic events occurred in three patients (8%) and nine patients (23%), respectively. A 3-year follow-up analysis of the ZUMA-12 trial results has also been reported [62]. After a median follow-up of 47.0 months, the CR rate increased to 86%, while the ORR was 92%. The estimated 36-month duration of response and EFS, PFS, and OS were 81.8%, 73.0%, 75.1%, and 81.1%, respectively. On the basis of the promising results of the ZUMA-12 trial, the phase III ZUMA-23 trial (NCT05605899) was designed to compare axi-cel with standard-of-care therapy in approximately 300 adult patients with LBCL, including those with DLBCL, high-grade B-cell lymphoma, and transformed follicular or marginal zone lymphoma, with IPI scores of 4–5 [63]. Patients in the axi-cel arm undergo leukapheresis followed by bridging therapy with R-CHOP or DA-EPOCH-R before treatment with lymphodepleting chemotherapy. Although the role of CAR-T-cell therapy in a first-line setting remains to be established pending the results of ZUMA-23, the development of cellular immunotherapy for use in earlier treatment lines has rapidly advanced.

### Therapeutic approach for high-risk central nervous system relapse

Several risk factors for central nervous system (CNS) relapse, including a high CNS-International Prognostic Index (CNS-IPI), involvement of specific extranodal sites (e.g., testis, breast, kidney, adrenal gland, bone marrow), and ≥ 3 extranodal sites, in newly diagnosed DLBCL have been identified. Molecular features such as ABC/unclassified subtype, CD5 positivity, and double-expressor lymphoma have also been associated with increased risk for CNS relapse. However, no optimal treatment regimen for the prevention of CNS relapse has been established. In clinical practice, the intrathecal (IT) administration of methotrexate (MTX) or cytarabine has been widely adopted for CNS prophylaxis. However, strong evidence that IT administration of these drugs is effective for the prevention of CNS relapse is lacking. Large systematic reviews and retrospective analyses have shown that IT is not an independent factor for the prevention of CNS relapse. High-dose MTX (HD-MTX), which is used for primary CNS lymphoma and as a component of intensive regimens, has also been investigated, but its benefit remains controversial. Recent prospective phase II trials from Japan that have incorporated HD-MTX in a sandwich approach with chemotherapy have reported relatively low CNS relapse rates in high-risk DLBCL patients, including those with CD5-positive DLBCL (PEARL5) [64] and intravascular large B-cell lymphoma (PRIMEUR-IVL) [65]. However, evidence remains limited to phase II studies. A randomized phase III JCOG2201 trial is currently ongoing and seeks to compare Pola-R-CHP plus IT therapy with Pola-R-CHP plus HD-MTX and IT therapy in patients with high-risk DLBCL [66].

### Therapeutic approach for limited-stage DLBCL

For limited-stage DLBCL, combined-modality therapy, including radiotherapy, has been widely studied. In the SWOG S8736 trial, compared with eight cycles of CHOP, three cycles of CHOP followed by involved-field radiotherapy (IFRT) improved PFS and OS in patients with stage I–nonbulky II aggressive lymphoma, although long-term follow-up did not differ between the two strategies [67–69]. The phase II S0014 trial evaluated three cycles of R-CHOP plus one cycle of rituximab followed by IFRT (40–46 Gy) in patients with low-risk limited-stage disease and reported favorable outcomes [70]. However, the role of radiotherapy, particularly for extranodal or bulky disease, remains controversial. In the UNFOLDER trial, consolidation radiotherapy improved EFS but not PFS or OS [71].

Patients with limited-stage DLBCL generally have excellent survival outcomes, and treatment strategies have increasingly focused on reducing toxicity (Table 4). The phase III FLYER trial compared six cycles of R-CHOP with four cycles of R-CHOP plus two additional cycles of rituximab in patients aged 18–60 years, with stage I-II, nonbulky disease (< 7.5 cm) and an IPI of 0 [72]. At a median follow-up of 66 months, the 3-year PFS was 96% for the 4-cycle group and 94% for the 6-cycle group, which demonstrates the noninferiority of the four-cycle group compared with the six-cycle group as well as reduced toxicity. These results established four cycles of R-CHOP with 2 cycles of rituximab as a standard treatment for younger patients with limited-stage DLBCL with no risk factors.

**Table 4:** Clinical trials with newly diagnosed limited-stage DLBCL in the rituximab era

Trial (References)	Phase	Number (limited stage)	Trial	Patients	Age, median	Outcomes
SWOG 0014 [70]	II	60 (60)	R-CHOP × 3 + R × 1 followed by IFRT (40–46 Gy)	CD20-positive aggressive lymphoma Age ≥ 18 yr ECOG PS 0–2 Stage-modified IPI 0–1	69	4-yr PFS: 88% 4-yr OS: 92%
UNFOLDER DSHNHL [71]	III	467 (302)	2 × 2 factorial design: R-CHOP14 × 6 + RT vs. R-CHOP14 × 6 R-CHOP21 × 6 + RT vs. R-CHOP21 × 6	Aggressive B-cell lymphoma Age 18–60 yrs aa IPI 0 and bulky disease (≥ 7.5 cm) or aa IPI 1	47	RT vs. observation 3-yr EFS: 84% vs. 68% 3-yr PFS: 89% vs. 81% 3-yr OS: 93**%** vs. 93%
FLYER DSHNHL [72]	III	588 (583)	R-CHOP × 6 vs. R-CHOP × 4 + R × 2	CD20-positive B-cell lymphoma Age 18–60 yrs ECOG PS 0–1 Non-bulky disease (< 7.5 cm) Aa IPI 0	6-cycles group: 47 4-cycles group: 49	6-cycles group vs. 4-cycles group 3-yr PFS: 94% vs. 96% 3-yr EFS: 89% vs. 89% 3-yr OS: 98% vs. 99%
SWOG S1001 [73]	II	60 (60)	R-CHOP × 3 followed by iPET-negative: R-CHOP × 1 iPET-positive: IFRT plus ibritumomab tiuxetan	CD20-positive aggressive lymphoma Stage I–II ECOG PS 0–2 Stage-modified IPI ≥ 1	69	2-yr PFS: 93% 4-yr PFS: 88% 2-yr OS: 93% 4-yr OS: 92%
Shi et al. China [74]	III	287 (287)	R-CHOP × 4 followed by R × 4 vs. R-CHOP × 2 + R × 2	DLBCL (achieving CR by R-CHOP x 4) Age 14–75 yrs Stage I–II IPI 0–1 Non-bulky disease (< 7.5 cm)	55	6-cycles group vs. 4-cycles group 2-yr PFS: 94% vs. 95% 2-yr EFS: 94% vs. 94% 2-yr OS: 99% vs. 99%
LNH2009-1B LYSA [75]	III	650 (616)	R-CHOP × 2 followed by iPET-negative: R-CHOP × 2 iPET-positive: R-CHOP × 4	DLBCL Age 18–80 yrs aa-IPI 0–1	NA	5-yr PFS: 87%, 5-yr OS 89% iPET-positive vs. iPET-negative 5-yr PFS: 86% vs. 89% 2-yr OS: 85% vs. 91%

aa IPI, age-adjsted International Prognostic Index; CHOP, cyclophosphamide, doxorubicin, vincristine, and prodnisone; DSHNHL, EFS, even-free survival; German High-grade Non-Hodgkin’s Lymphoma Study Group; IFRT, involved-field radiation therapy; iPET, interim positron emission tomography; NA, not available; OS, overall survival; PFS, progression-free survival; R, rituximab; RT; radiation therapy; SWOG, Southwest Oncology Group; vs., versus; yr, year

PET-adapted strategies to reduce chemotherapy exposure in limited-stage DLBCL have been investigated. In the SWOG phase II S1001 trial, patients with stage I-II and nonbulky disease (< 10 cm) received three cycles of R-CHOP followed by PET-guided therapy; patients with negative interim PET scans received one additional cycle of R-CHOP, whereas PET-positive patients received involved-field radiotherapy plus ibritumomab tiuxetan [73]. The outcomes were similar in the PET-negative and PET-positive groups. A Chinese phase III trial demonstrated that four cycles of R-CHOP plus four cycles of rituximab was noninferior to six cycles of R-CHOP plus two cycles of rituximab in patients who achieved PET-confirmed CR after four cycles, with reduced toxicity [74]. Similarly, the phase III LYSA LNH2009-1B trial revealed that a PET-adapted strategy that allowed four cycles of R-CHOP in patients with negative interim PET scans was noninferior to the standard six cycles and was associated with fewer severe adverse events [75]. These studies support the use of PET-adapted treatment with abbreviated chemotherapy as a standard approach for low-risk limited-stage DLBCL.

## Conclusion

Significant progress has been made in the development of novel treatments for newly diagnosed DLBCL. Advances in basic research have led to the incorporation of targeted agents into conventional chemotherapy regimens. In addition, cellular therapies such as CAR-T-cell therapy and bispecific antibodies have improved outcomes in R/R DLBCL patients and are now under investigation in frontline settings. Chemo-free approaches are being actively developed for DLBCL. However, their efficacy in the frontline setting remains to be established, and even if positive, they are unlikely to immediately replace established cytotoxic chemotherapeutic regimens. Long-term follow-up is required to determine whether these strategies can achieve cure. Treatment approaches will increasingly move toward molecularly or subtype-based individualized therapy. Currently, there is a lack of standardized molecular stratification. For successful implementation in routine clinical practice, there is a critical need for widely accessible, cost-effective, and rapid methods to identify relevant molecular targets and subtypes.
